# Graphene Nanoplatelets for the Development of Reinforced PLA–PCL Electrospun Fibers as the Next-Generation of Biomedical Mats

**DOI:** 10.3390/polym12061390

**Published:** 2020-06-21

**Authors:** Enrica Chiesa, Rossella Dorati, Silvia Pisani, Giovanna Bruni, Laura G. Rizzi, Bice Conti, Tiziana Modena, Ida Genta

**Affiliations:** 1 Department of Drug Sciences, University of Pavia, V.le Taramelli 12—27100 Pavia, Italy; enrica.chiesa@unipv.it (E.C.); rossella.dorati@unipv.it (R.D.); bice.conti@unipv.it (B.C.); tiziana.modena@unipv.it (T.M.); 2 Polymerix srl, V.le Taramelli 24—27100 Pavia, Italy; 3 Immunology and Transplantation Laboratory, Pedriatric Hematology Oncology Unit, Department of Maternal and Children’s Health, Fondazione IRCCS Policlinico S. Matteo—27100 Pavia, Italy; silvia.pisani01@universitadipavia.it; 4 Department of Chemistry, Physical Chemistry Section, University of Pavia, Via Taramelli 12/14, 27100 Pavia, PV, Italy; giovanna.bruni@unipv.it; 5 Directa Plus S.p.a., COMO NexT, Via Cavour, 2—22074 Lomazzo (CO), Italy; laura.rizzi@directa-plus.com

**Keywords:** electrospinning, graphene nanoplatelets, biodegradable polymers, poly-l-lactide-co-poly-ε-caprolactone, composite scaffolds

## Abstract

Electrospun scaffolds made of nano- and micro-fibrous non-woven mats from biodegradable polymers have been intensely investigated in recent years. In this field, polymer-based materials are broadly used for biomedical applications since they can be managed in high scale, easily shaped, and chemically changed to tailor their specific biologic properties. Nonetheless polymeric materials can be reinforced with inorganic materials to produce a next-generation composite with improved properties. Herein, the role of graphene nanoplatelets (GNPs) on electrospun poly-l-lactide-co-poly-ε-caprolactone (PLA–PCL, 70:30 molar ratio) fibers was investigated. Microfibers of neat PLA–PCL and with different amounts of GNPs were produced by electrospinning and they were characterized for their physicochemical and biologic properties. Results showed that GNPs concentration notably affected the fibers morphology and diameters distribution, influenced PLA–PCL chain mobility in the crystallization process and tuned the mechanical and thermal properties of the electrospun matrices. GNPs were also liable of slowing down copolymer degradation rate in simulated physiological environment. However, no toxic impurities and degradation products were pointed out up to 60 d incubation. Furthermore, preliminary biologic tests proved the ability of the matrices to enhance fibroblast cells attachment and proliferation probably due to their unique 3D-interconnected structure.

## 1. Introduction

Electrospinning has been revealed a relatively simple, robust and up-scalable technique at the forefront of micro-nanofiber mats fabrication for industrial (e.g., water/air filtration, textile and packaging materials, optical electronics and biosensors) and biomedical/pharmaceutical applications (e.g., tissue engineering and regenerative medicine, wound dressing, implant coating films and in-product labeling of tablets, drug delivery) [1,2,3,4,5,6,7,8,9,10,11,12]. This process offers unique capabilities for producing nanofibrous fabrics with wide variety of sizes and shapes, extremely high surface-to-volume ratio and tunable porosity [1,13]. The feasibility lies into the possibility of properly tuning process parameters such as voltage, feed flow rate, needle tip-collector distance, collector and spinneret type as long as selecting suitable raw materials and their solutions concentration in suitable solvents, viscosity and conductivity [14].

Aliphatic polyester-based polymers are an attractive class of materials for manufacturing fibrous mats with a broad spectrum of biomedical applications and among them, poly-lactic acid (PLA) and poly-ε-caprolactone (PCL) have fueled technological and commercial interest since both polymers are biocompatible, biodegradable, resorbable and FDA-approved for clinical use in humans [15,16,17,18,19,20,21]. Depending on their enantiomeric purity, PLAs are semicrystalline/amorphous, stiff and brittle polymers derived from renewable resources, with a relatively high glass transition temperature (55–70 °C) and characterized by a low thermal conductivity and high tensile strength [22]. On the contrary, PCL is a highly rubbery, ductile polymer with a Tg around −60 °C exhibiting low mechanical strength, good toughness and slow degradation rate (two-four years depending on the starting molecular weight) due to its high hydrophobicity [23]. PLA/PCL blend-based electrospun fibers have been widely investigated for broadening their range of use [24,25]. Nonetheless, the PLA/PCL blends thermodynamic immiscibility and their poor interfacial adhesion cause unreliable electrospinning process and compromise fiber mat quality. For these reasons, several improvement strategies are required such as: (*i*) addition of polymeric compatibilizers, commonly copolymers made of blocks identical or similar to blend components, (*ii*) induction of newly formed grafted copolymers by chemical reaction among blend components functional groups during the blend melt-mixing (reactive compatibilization) or (*iii*) nanofillers addition [26].

Graphene is an allotrope form of carbon consisting of a single layer of sp^2^-hydridized carbon atoms arranged in a 2D honeycomb lattice. This inert compound and its derivatives gained great interest as environmentally friendly materials for different industrial uses owing to their unique properties (e.g., excellent electrical and thermal conductivity, high mechanical strength, wide surface area and easy chemical functionalization). Moreover, graphene and its derivatives were extensively investigated as nanofillers for neat PLA, PCL and PLA/PCL blend fibers leading to a good combination of electrical, mechanical and thermal properties, and more recently, graphene nanoderivatives resulted to be promising for biomedical applications including drug and gene delivery, biosensors and molecular imaging, tissue engineering and regenerative medicine [27,28,29,30,31]. To date, the positive effect of graphene nanoderivatives on PLA/PCL blend electrospun fibrous mats was evaluated for graphene nanoplatelets (GNPs), on matrices obtained by melt-spinning technique, and for graphene oxide or multiwall carbon nanotubes on electrospun matrices. In all cases graphene derivatives addition demonstrated simultaneous reinforcement and enhancement of biologic properties [32,33,34].

Starting from this background, in this work the effect of GNPs loading into electrospun fiber mats made from preformed polylactide-co-polycaprolacton block copolymer (PLA–PCL) was investigated with the following hypothesized advantages: PLA–PCL is arranged with reproducible molecular weight and desirable PLA- and PCL-blocks composition to ensure tunable mechanical/thermal behavior and degradation rate; electrospinning procedure takes place under conditions milder than melt-spinning technique and it is more suitable for loading labile drugs such as growth factors. Final aim of this study was the design and development of next-generation polymeric electrospun matrices loaded with GNPs (EL_GNPs) for biomedical use.

In a previous work we had focused on identifying those process and formulation parameters critical to setup an electrospinning procedure in order to obtain PLA–PCL fibrous mats with tunable features [35]. In the current study, newly synthetized pristine GNPs were selected as reinforcement agents endowing PLA–PCL fiber mats with special functions for advanced applications. GNPs (C/O ratio > 200) were obtained by an ecofriendly continuous process involving expansion process at high temperature followed by exfoliation and size reduction treatments by ultrasounds or high pressure homogenization and they were introduced as concentrated, aqueous dispersion (without any surfactants), easy-to-use, safe from a health and environmental point of view [36]. The influence of the GNPs amount on fibers morphology and crystallization behavior, in vitro degradation profile, mechanical strength and thermal conductivity was analyzed in this work and preliminary biologic studies were addressed to ensure EL_GNPs as suitable support for cell migration and proliferation from surrounding tissues.

## 2. Materials and Methods

### 2.1. Materials

Poly-lactic acid-co–poly-ε-caprolactone copolymer (PLA–PCL, Resomer^®^ LC 703 S Esther terminated-lactide:caprolactone 70:30 molar ratio, M_w_ 140–160 kDa) was purchased from Evonik Industries (Evonik Nutrition & Care GmbH, 64,275 Damstadt, Germany). Pristine graphene nanoplatelets (GNPs), with thicknesses 2–4 nm and lateral dimension up to a 10–15 μm, were kindly gifted by Directa Plus S.p.A. (Lomazzo, Italy) [36]. Methylene chloride (MC), *N,N*-dimethylformamide (DMF) and tetrahydrofuran (THF) analytical grade were supplied by Carlo Erba (Milan, Italy) and used without further purification. Dulbecco’s Modified Eagle’s Medium—high glucose (DMEM), Dulbecco’s phosphate buffered saline (PBS 10X, sterile), penicillin–streptomycin solution (100X), dimethyl sulfoxide (DMSO), 3-(4,5-dimethylthiazol-2-yl)-2,5-diphenyltetrazolium bromide and Hoechst 33,258 solution were obtained from Sigma Aldrich (St. Louis, MO, USA). Normal human dermal fibroblasts (NHDFs) were provided by PromoCell (Heidelberg, Germany); the cells were cultured at 37 °C with 5% CO_2_ in 25 cm^2^ culture flask with DMEM containing 1% (*v/v*) penicillin–streptomycin and 10% (*v/v*) FBS.

### 2.2. Electrospinning Process

The electrospun matrices were prepared by using a GMP-oriented electrospinning apparatus Nanon-01A (MEEC Instruments, Ltd., Ogori-shi, Fukuoka, Japan) combined to a dehumidifier (MEEC instruments, MP, Pioltello, Italy). The instrument consists of three main components: a high potential voltage generator, a spinneret (a metal part connected to the voltage generator) and a flat metal collector recovering the sample. The polymeric solution is loaded in a syringe, on that a constant pressure was applied by using a mechanical pump. On the basis of our previous works [10,35,37], the electrospinning parameters were 20 kV voltage, 0.1 mL/h flow rate, and 15 cm between the needle (ID 18G) and the collector.

PLA–PCL was dissolved in MC to reach a final concentration of 28.5% *w/v*. Increasing GNPs amounts were homogenously suspended under vigorous agitation for 1 h in DMF and then GNPs suspension was added to the polymeric solution under magnetic stirring for 3 h at room temperature: the MC:DMF volumetric ratio was previously set at 70:30 so the calculated final PLA–PCL concentration in the solvent mixture was 20% *w/v* [35]. Several GNPs final concentrations were tested, namely 0.5 wt %, 1 wt %, 2 wt % compared to the polymer amount (GNPs_0.5%, GNPs_1%, GNPs_2%, GNPs_4%).

The electrospinning process was carried out for 20 min at 30 ± 2.5 °C and 25% ± 5% relative humidity.

The resultant neat and composite mats (Table 1) were left in a climatic chamber at 37 °C for 48 h to facilitate the removal of solvent traces and then stored in zip bags in a desiccator.

### 2.3. Characterization of PLA–PCL/GNPs Blends

To evaluate GNPs dispersion in PLA–PCL solution and possible aggregates formation, copolymer blends containing different amounts of GNPs (GNPs_0.5%, GNPs_1%, GNPs_2%, GNPs_4%) were observed by using an optical microscope (Carl Zeiss, Axio LabA1 HAL, condenser 0.9/1.25 H, objective ICS A PLAN 63x/0.80 PH2, IDS camera UI-3260CP-M-GL Rev.2).

The rheological behavior of PLA–PCL solution (20% *w/v*) in MC:DMF (70:30 *v/v*) (GNPs_0%) and of the blends of PLA–PCL with GNPs at different concentration, prepared as reported above, were detected by a rotational rheometer Kinexus Plus (Malvern, Alfatest, Milano, Italy) equipped with a temperature control system. Data processing was recorded with the rSpace software. A cone–plate system (1° cone angle, diameter 40 mm) with a gap of 0.15 mm was used.

First, the viscosity was assessed at increasing shear rate from 0.1 to 10 s^−1^ (shear rate ramp test). In particular, the viscosity value was determined at the specific shear rate, applied to polymeric solution when eluted through the needle during electrospinning process. The shear rate value was calculated by the following equation [38,39]:

γ′ = 4Q/(πr^3^) (1)

where γ′ is the sliding gradient applied on the needle wall, Q is the volumetric flow rate and r is the needle radius (internal diameter = 0.840 mm; external diameter = 1.2 mm). The resulted shear rate (γ′) was 0.5 s^−1^.

Amplitude sweep test was carried out to evaluate the linear viscoelastic region for each sample and then frequency sweep test was performed to measure the viscoelastic response of samples at 25 °C in an oscillation mode (10–0.1 Hz), at constant stress (0.1 Pa). Polymer solution viscoelastic properties were assessed by measuring storage modulus (G′) and loss modulus (G″) representing the elastic behavior and viscous behavior of the materials, respectively.

### 2.4. Electrospun Matrices Characterization

#### 2.4.1. Morphology

Morphometric analysis of electrospun fiber mats was carried out by scanning electron microscope (SEM) Zeiss EVO MA10 (Carl Zeiss, Oberkochen, Germany). The samples were gold-sputtered under argon before to be undergone to the analysis. Microphotographs at two different magnifications were collected: 3-kX and 10-kX. SEM images were processed by ImageJ software (ImageJ 1.52a, U. S. National Institutes of Health, Bethesda, MD, USA) and fibers diameter analyzed through ImageJ diameter plugin.

#### 2.4.2. Differential Scanning Calorimetry Analysis

Crystallization behavior of neat and composite matrices was studied by differential scanning calorimetry (DSC, DSC Q2000 apparatus interfaced with a TA 5000 data station, TA Instrument, New Castle, DE, USA). Samples (2–4.5 mg) were hermetically sealed in aluminum pans. Cyclic measurements under nitrogen flow (50 mL/min), with a first heating to 180 °C at the heating rate of 5 K/min, cooling down to 20 °C at 5 K/min and a second heating up to 180 °C at 2 K/min were taken. The first heating was performed in order to eliminate the effect of thermal and processing history. Temperature and enthalpy change of the cold crystallization (T_cc_, ΔH_cc_) and of the melting (T_m_, ΔH_m_) for neat and composite mats were obtained from the DSC curve during the second heating.

### 2.5. Biologic Study: Cell Adhesion and Proliferation on Electrospun Matrices

50,000 NHDFs were seeded on the electrospun matrices and incubated at 37 °C, 5% of CO_2_ for 3 h and up to 168 h (7 d). Circular shape specimens of 1.8 cm^2^ area were used for this experiment. Cell medium was refreshed every 48 h along all test time. Untreated cells (without mat support) and pristine GNPs, amounting to the same weight of graphene contained into each composite mat sample, were used as controls.

At scheduled times (3 h, 72 h and 168 h), the samples were collected, and cell viability was determined by MTT assay. Results were expressed as absorbance at 570 nm and then cell viability percentage was calculated compared to the untreated cells (CTR+). At each time point, samples were quickly washed with PBS and nuclei were counterstained for DNA with 0.5-μg/mL Hoechst 33258. Samples were observed by means a Zeiss Axiophot fluorescence microscope (Carl Zeiss, Oberkochen, Germany (blue filter: λex = 346 nm and λem = 460 nm; green filter: λ = 494 nm and λem = 518 nm). All samples were exposed to UV light overnight before incubation with cell cultures.

### 2.6. In Vitro Degradation Study

The electrospun matrices degradation behavior was evaluated in simulated physiologic condition and it was performed as follows: round-shaped composite mats samples (1.8 cm^2^) were incubated in 5 mL of DMEM with FBS (10% *v/v*) and antibiotics (1% *v/v*) at 37 °C up to 7, 14, 21, 28 and 60 d. At each scheduled time point the incubation medium was withdrawn, and the electrospun matrices were collected and characterized for their morphology (Section 2.4.1), fluid uptake and mass loss (Section 2.6.1); PLA–PCL molecular weight was determined by using Gel Permeation Chromatography (GPC) (Section 2.6.2). Samples of the incubation media were further incubated with NHDFs to check the presence of potentially toxic impurities and degradation products of the electrospun matrices (Section 2.6.3).

#### 2.6.1. Fluid Uptake and Mass Loss

Electrospun matrices were weighted (M_0_) and then incubated at 37 °C in a thermostated water bath with 5 mL of DMEM with FBS and antibiotics. At the scheduled time points 7, 14, 21 and 28 d the mats were collected, washed three times in order to remove any residual of culture medium and weighted wet (M_t_) immediately after wiping the surface with a filter paper to adsorb the exceeding fluid. The samples were freeze dried overnight (−48 °C, 0.02 bar) (Lio5P, 5Pascal, Milano, Italy) and the freeze-dried samples weighted (M_x_). Fluid uptake (FU) percentage and mass loss percentage were calculated according to Equations (2) and (3), respectively:

Fluid uptake (%) = 100 × (M_t_ − M_0_)/M_0_ (2)

Mass loss (%) = 100 × (M_0_ − M_x_)/M_0_ (3)

The test was performed in triplicate for each specimen and results were reported as mean ± standard deviation (SD).

#### 2.6.2. PLA–PCL Molecular Weight Determination

PLA–PCL molecular weight determination was performed by 1260 Infinity GPC apparatus (Agilent Technologies, Santa Clara, CA, USA) equipped with three different PhenoGEL columns (5-µm particle size—500-Å, 103-Å, 104-Å pore size) and a refractive index detector. THF was used as mobile phase and flow rate was set at 1 mL/min.

Electrospun matrices were dissolved in THF to reach the final PLA–PCL concentration of 3 mg/mL, GNPs was removed by centrifugation and the polymer solutions were filtered throughout 0.45 μm nylon filters and analyzed. PLA–PCL molecular number, molecular weight and polydispersity index were determined against polystyrene standards whose molecular weight ranged from 1480 and 361,500 Da. Neat PLA–PCL mats were used as control.

#### 2.6.3. Cytotoxicity of Mats Degradation Products

With the aim of assessing the presence of toxic products/impurities released from the mats, a cytotoxicity test was performed on the incubation media recovered by in vitro degradation experiments (see Section 2.6).

1 × 10^4^ NHDFs were seeded into 96-well plate and incubated at 37 °C and 5% of CO_2_ for 24 h. Afterwards, cell medium was withdrawn and 200 μL of incubation medium from in vitro degradation experiments were added; the samples were incubated for 24 h at 37 °C and 5% CO_2_. Cells cultured fresh cell medium (200 μL) were used as positive control (CTR+). Cell viability was assessed by MTT assay. Results were expressed a cell viability percentage compared to the CRT+.

### 2.7. Mechanical Properties

Uniaxial tensile tests were carried out by an electromagnetic tensiometers (Electromagnetic MTS Insight System 10 kN, MTS System Corporation, Eden Prairie, MN, USA) equipped with a video extensometer NG (Messphysic Material Testing) and a 250-N load cell.

Neat and composites mats (*n* = 3) were prepared as described in Section 2.2 and electrospinning process was carried on for 3 h to achieve a suitable mat thickness. The tensile test was carried out at constant speed of 10 mm/s and on dog-bone shaped specimens of 25-mm length, 5-mm width and 0.12-mm thickness (four replicates for each mats) according to ASTM D638.

The results are reported as stress values and Young’s modulus.

### 2.8. Thermal Conductivity

Thermal conductivity and thermal diffusivity, hence the specific heat capacity per unit volume of material, were evaluated on selected composite mats accordingly to ISO 22007-2:2015 through the transient plane heat source (hot disc) method (TPS 3500, Hot Disk AB, Gothenburg, Sweden). The analyses were carried out at 25 °C, 62% R.H. and repeated in triplicate for each type of electrospun matrix.

### 2.9. Statistical Analysis

All experiments were carried out in triplicate unless otherwise stated. All results were presented as the mean and standard deviation (SD). Statistical analysis tool was performed by GraphPad Prism 6 software (Graphpad software, La Jolla, CA, USA) using one-way analysis of variance (ANOVA) with either Sidak’s or Tukey’s multiple comparison analysis.

## 3. Results

### 3.1. Characterization of PLA–PCL/GNPs Blends

**Figure 1:**
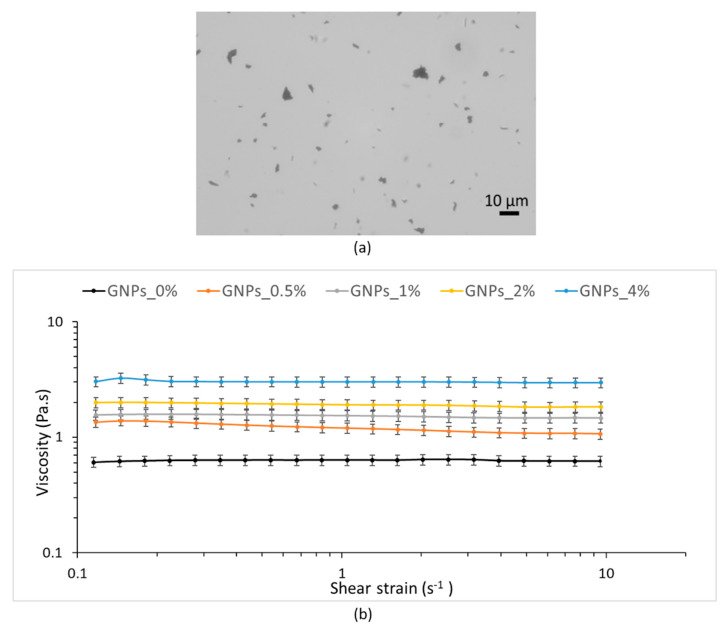
(**a**) Optical microscope representative image of GNPs_2%; (**b**) shear viscosity of PCL–PLA solution (GNPs_0%) and GNPs_0.5%, GNPs_1%, GNPs_2% and GNPs_4% blends in methylene chloride (MC): *N,N*-dimethylformamide (DMF) (70:30 *v/v*).

Nanofiller dispersion into polymer solution is crucial to the final properties composite mats [33]. For this reason, PLA–PCL/GNPs blends were observed by optical microscope. Figure 1a shows, as an example, the optical microscope image of GNPs_2%: GNPs revealed sizes ranging from few to about 10 μm confirming the same GNPs lateral dimensions as declared by the manufacturer.

Considering the importance of polymer mixtures rheological properties for obtaining homogeneous electrospun matrices with uniform fibers, the rheological behavior of both neat PLA–PCL solution in MC:DMF (GNPs_0%) and copolymer mixtures containing different amounts of GNPs was studied. During electrospinning process, electrostatic forces of the applied voltage should counteract the viscoelastic stresses and solution surface tension, thereby shear rheological properties of a solution can help to understand its flow behavior inside the needle during electrospinning process where mainly elongational flow occurs [40,41]. If shear viscosity η is high, resistance to flow also will be high; therefore, keeping applied voltage constant, nanofiber’s diameter will increase as viscosity increases.

The results of amplitude sweep test carried out at a constant frequency of 1 Hz, with a shear stress of 0.1–10 Pa, showed that the viscoelastic region resulted to be between 0.1 and 0.177 Pa for the PLA–PCL solution, 0.1 and 0.194 Pa for the GNPs_1%, 0.1 and 0.375 Pa for the GNPs_2% and 0.1 and 0.475 Pa for the GNPs_4%.

Starting from the data of shear stress included in the linear viscoelastic region, dynamic viscoelasticity was calculated by frequency sweep test and provided the viscoelastic properties of PLA–PCL at 25 °C by measuring the storage modulus and the loss modulus as a function of the oscillatory frequency, 0.1–10 Hz. PLA–PCL solution and the mixtures with GNPs at increasing concentrations displayed a loss modulus that was always higher than storage modulus over the entire frequency range. The most remarkable difference between storage modulus and loss modulus was observed at lower frequencies and both moduli increased with increasing frequencies (data not reported).

Next, shear rate ramp test runs were assessed in the shear rate range of 0.1–10 s^−1^ and viscosity value was measured. All samples tested showed Newtonian behavior over this range of shear rates; however, it should be noted that shear thinning will occur at higher shear rates. Figure 1b plots the viscosity as a function of shear rate and shows how the increase of GNPs concentration triggered a significant viscosity enhancement. Dynamic viscosity values determined at 0.5 s^−1^ are reported because the fixed 0.5 s^−1^ shear rate value represents the shear rate applied to the polymeric solution eluted through the needle during the electrospinning process. The values showed significant increases from 0.63 Pa.s for the neat PLA–PCL solution to 1.25 Pa.s for the blend GNPs_0.5%, and to 1.55 Pa.s, 1.93 Pa.s and 3.02 Pa.s with further increasing of GNPs concentration to 1 wt %, 2 wt % and 4 wt %, respectively. Indeed, by adding GNPs to the PLA–PCL up to the concentration of 4 wt % the solution dynamic viscosity was five time greater.

### 3.2. Electrospun Matrices Characterization

**Figure 2:**
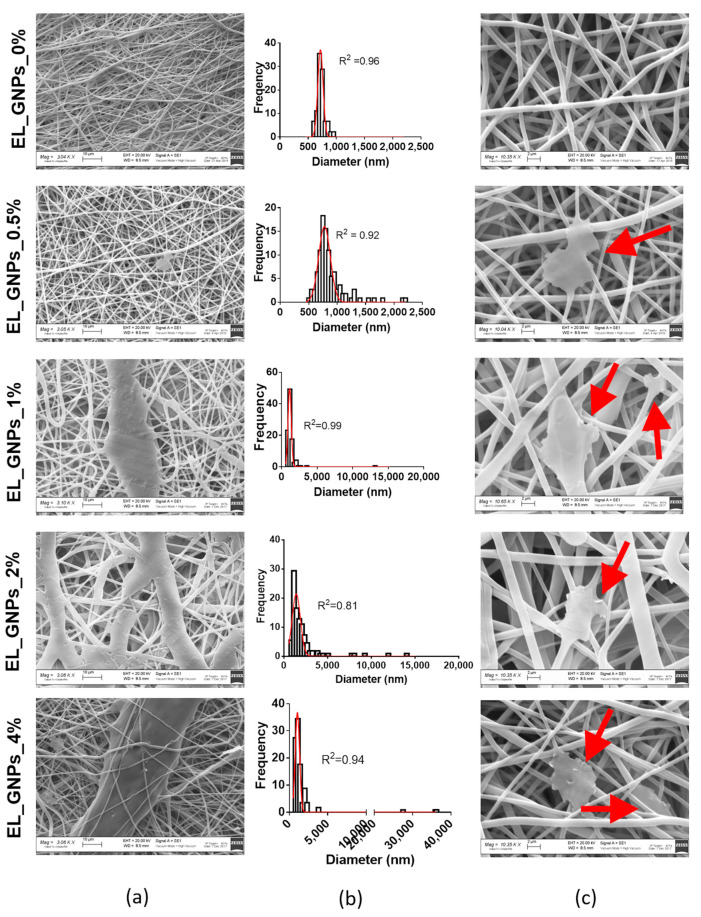
(**a**) SEM microphotographs (3 kX magnification, scale bar 10 μm) of EL_GNP_0%, EL_GNP_0.5%, EL_GNP_1%, EL_GNP_2% and EL_GNP_4% mats; (**b**) diameter distribution of the fibers which fitted with the gaussian curve with an R square value higher than 0.80; (**c**) SEM micrographs (10 kX magnification, scale bar 2 μm) of EL_GNP_0%, EL_GNP_0.5%, EL_GNP_1%, EL_GNP_2% and EL_GNP_4% mats: red arrows point out GNPs positioning.

Neat EL_GNP_0% morphometric analysis performed by SEM analysis, combined with ImageJ diameter plugin processing, showed homogeneous fibrous matrix with mean diameter 717 ± 46.8 nm and porosity suitable to mimic physiologic environment, as already demonstrated in our previous study [7,33] and shown in Figure 2a. Mean diameter gradually increases from 776 ± 102.7 nm (EL_GNPs_0.5%) to 1129 ± 224.2 nm (EL_GNPs_1%), 1407 ± 476.1 nm (EL_GNPs_2%) and 1053 ± 278.2 nm (EL_GNPs_4%), as predicted by viscosity measurements of the respective copolymer/GNPs mixtures.

It is also evident from Figure 2b that GNPs addition was responsible for fibers heterogenicity, since broader diameters gaussian distributions were observed increasing GNPs concentration from 0.5 wt % to 4 wt % and the percentage of fibers with a mean diameter higher than 2 μm consistently increased from 1.82% to 16.77%. The widest diameters distribution was observed for EL_GNPs_4% where the 1.6% of total fibers processed resulted in the highest mean diameter of about 35 μm, whereas no fiber with mean diameter higher than 2 μm resulted in polymeric mats without GNPs. This behavior can be explained by different GNPs positioning in the polymer fibers, either embedded or stacked on their surfaces. In the first case the fibers resulted homogeneous with diameter bigger than 2 μm, whereas GNPs positioned on polymer fibers surface, as shown by the yellow arrow in the SEM images enlargements (Figure 2c), resulted in not homogeneous enlargements of polymer fibers. Nevertheless, a parallel alignment of GNPs sheets in the fibers axis is evident, confirming the reliability of the electrospinning process in promoting parallel orientation of GNPs’ base plane to the flow direction, as already demonstrated by the literature for others graphene derivatives incorporated into PLA/PCL blended nanofibers [34,42].

#### Differential Scanning Calorimetry Analysis

**Figure 3:**
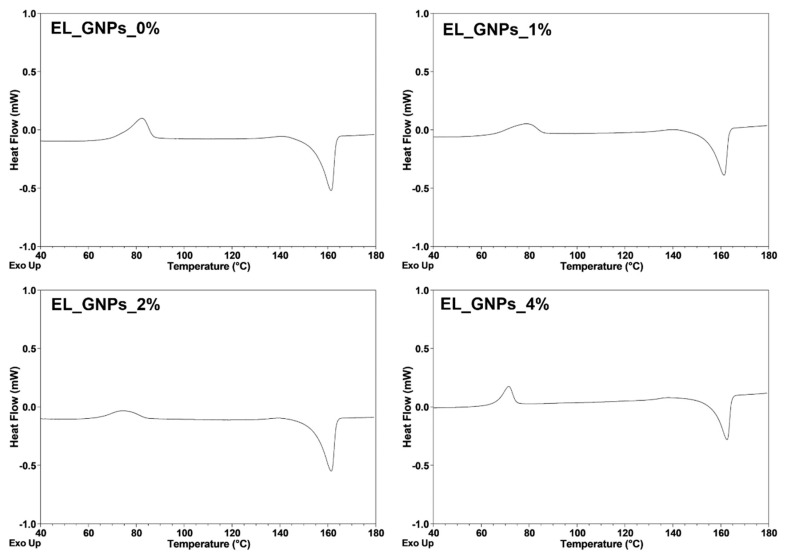
Representative second heating thermographs for EL_GNP_0% (**a**), EL_GNP_1% (**b**), EL_ GNP_2% (**c**) and EL_GNP_4% (**d**) matrices at a heating rate of 2 K/min.

Crystallization and melting behaviors of neat EL_GNPs_0% and composite EL_GNPs matrices were evaluated by DSC analysis. The DSC curves recorded during the second heating and the thermal parameters are shown in Figure 3 and Table 2, respectively.

Neat mats exhibit a unimodal endothermic melting peak at a temperature of ~156 °C suggesting the existence of PLA–PCL in crystalline arrangement. The lack of double melting peak indicates that only stable crystals of polymer were formed during the cold crystallization process due to the homogeneous nucleation mechanism of PLA–PCL. It can be observed that the melting peak of composite EL_PLA–PCL/GNPs matrices are also characterized by a unimodal endotherm. This may happen because the addition of GNPs in the polymer matrix led to formation of PLA–PCL crystals with uniform thickness. Further, the addition of the GNPs did not significantly change T_m_ which was kept unchanged at 161 °C. A cold crystallization exotherm is observed at the temperature of 66 °C for neat mats and in the temperature range of 64–72 °C in case of composite mats. Cold crystallization peak temperature (T_cc_) increased remarkably when GNPs were incorporated into electrospun composite mats (EL_GNPs_1%), but T_cc_ decrease was recorded in presence of high GNPs amounts with respect to the copolymer (EL_GNPs_2%–4%). This behavior suggests that GNPs could hinder or promote nucleation of PLA–PCL-based matrices as a function of their concentration inside the copolymer as previously documented for PLA- or PLA/PCL blend-based mats [28,33,43].

### 3.3. Biologic Study: Cell Adhesion and Proliferation on Composite Electrospun Matrices

Electrospun PLA–PCL-based fibrous mats cellularization was deeply investigated in our previous work [7] demonstrating that the cells adhered, proliferated and colonized patches, thus confirming the biocompatibility of the PLA–PCL-based fibrous mats.

Herein, the reduction activity of the MTT was measured after 3 h, 72 h (3 d) and 168 h (7 d) on NHDFs seeded on EL_GNPs composite scaffolds and reported as measurement of cell viability.

**Figure 4:**
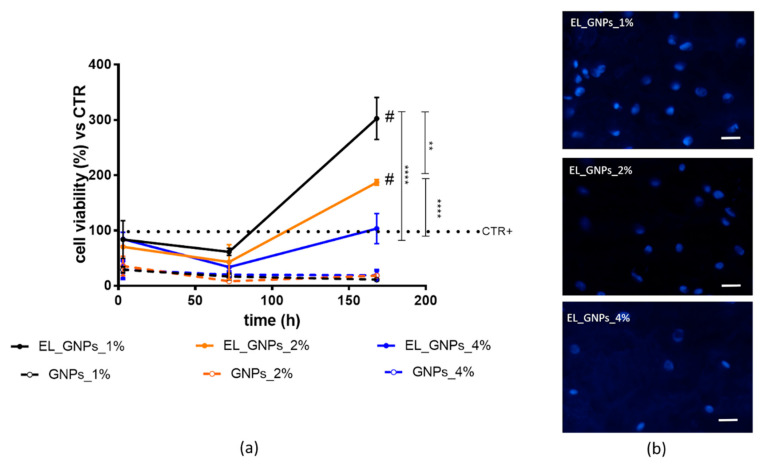
(**a**) Cell proliferation profiles up to 168 h (7 d) of incubation on electrospun matrices (EL_ GNP_1%, EL_GNP_2%, EL_GNP_4%) and co-incubated with equivalent amounts of free GNPs. Cells grown without support were chosen as control (CTR+). Results are expressed as cell viability percentage, mean ± SD (*n* = 3). Multiple comparison test revealed significant differences for (#) *p* value < 0.05, (**) < 0.01, (****) < 0.0001; (**b**) fluorescence microscopy images of fibroblasts seeded on electrospun composite matrices at 168 h (7 d). Blue fluorescence denotes nuclear DNA stained with Hoechst33258 dye (obj 20× magnification, scale bar = 20 µm).

As shown in Figure 4a, no significant differences were highlighted in cell adhesion after 3 h of incubation on EL_ GNP_1%, EL_GNP_2% and EL_GNP_4% disclosing a cell viability percentage of 83.69%, 70.37% and 84.39%, respectively. Therefore, fibrous structure of the electrospun matrices efficiently promotes cell adhesion; moreover, cell adhesion occurred preferentially on the fibrous matrices, with respect to the corresponding free GNPs which showed about 30% cell viability. This evidence was confirmed for the prolonged incubation times up to 72 h, showing cell proliferation on the electrospun matrices consistently higher than the cell proliferation on free GNPs. More in detail, after 72 h incubation a slight decrease in cell viability percentage was observed on the fibrous mats, if compared to the first 3 h of incubation (61.31%, 43.04% and 33.81% for EL_GNP_1%, EL_GNP_2% and EL_GNP_4%) and the decrease is consistent with higher amounts of GNPs. In particular, the lowest GNPs concentration revealed the best cell proliferation values (*p* value < 0.01, Tukey’s comparison test).

Finally, after 168 h of incubation, cell viability improved (*p* < 0.05) compared to the initial timing (3 h) and compared to CRT+ for EL_GNP_1% and EL_GNP_2%, as indicated by the hash (#) in Figure 4a. Instead, the cell proliferation for EL_GNP_4% was superimposable to cell control (cells without support, CTR+, dashed line, Figure 4a). EL_GNP_1% and EL_GNP_2% reached the maximum level of cell proliferation and showed 4-fold and 2-fold increase of cell viability if compared to CTR+, respectively. Figure 4b shows fluorescent microscope images of the cells seeded on the mats after 7 d of incubation. It can be observed that the mats had adequate surface for cell proliferation; after 7 d, high cellular density was observed for EL_ GNP_1%. However, in all the mats, fibroblasts showed cytoplasmic prolongations, which probably facilitated cellular adhesion and communication between cells. Furthermore, a significant difference in cell viability was highlighted between composite electrospun matrices and free GNPs.

### 3.4. In Vitro Degradation Study

Hydrolytic degradation of biodegradable (co)polyesters is affected by a great number of factors, such as chemical composition, hydrophilicity, pH of incubation medium, sample morphology, polymer molecular weights. More in details, the degradation behavior of electrospun matrices can be affected by additional factors as porosity, surface/volume ratio and manufacturing process.

**Figure 5:**
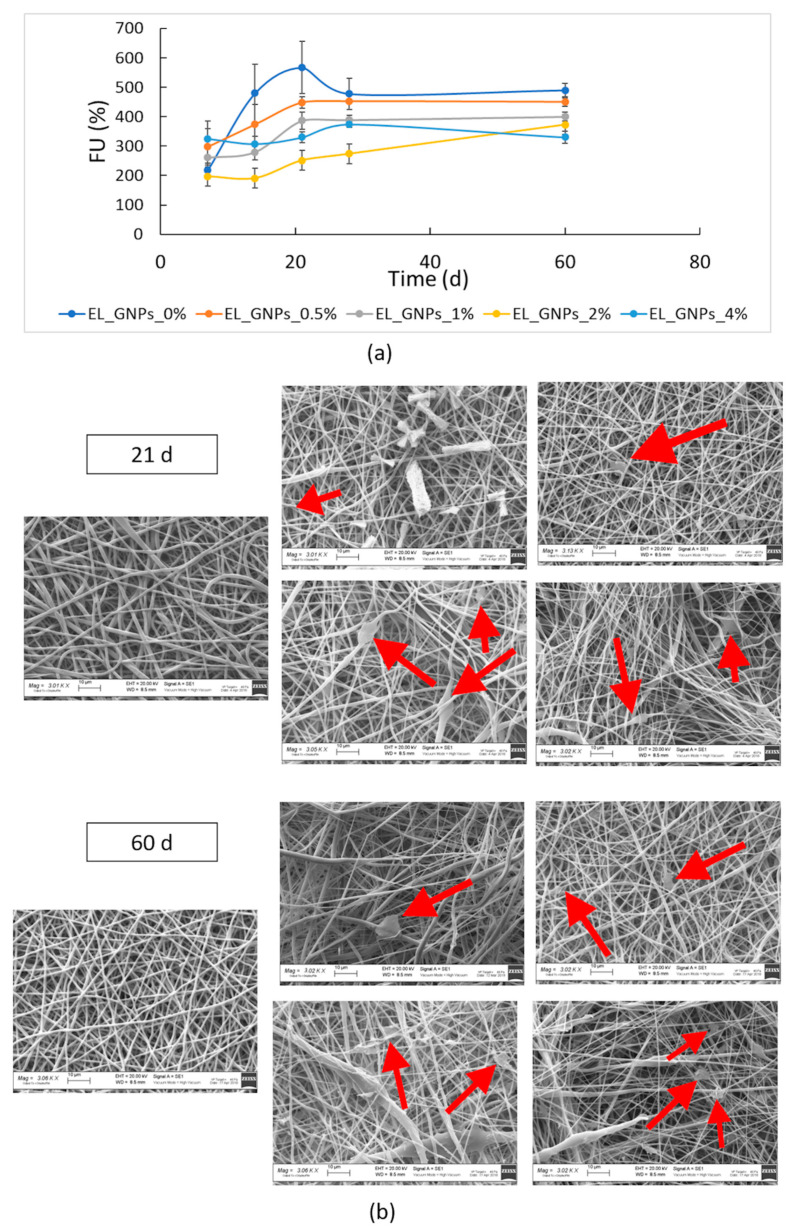
(**a**) Fluid uptake (FU) percentage of neat EL_GNP_0% and EL_GNPs composite matrices during 60 d of incubation at 37 °C in 5 mL of DMEM (10% FBS, 1% antibiotics); (**b**) SEM micrographs (3 kX magnification, scale bar 10 μm) of freeze-dried EL_GNP_0%, EL_GNPs composite matrices after 21 d and 60 d incubation.

Degradation study was performed incubating the electrospun matrices at 37 °C in DMEM with FBS and antibiotics up to 60 d. Fluid absorption, mass loss and changes in polymer molecular weight were evaluated. All samples showed high DMEM absorption (>200%) and Figure 5a clearly shows that fluid uptake value increased up to 21 d of incubation for EL_GNP_0%, EL_GNP_0.5% and EL_ GNP_1% while it kept constant throughout all incubation time for the samples with higher graphene content (EL_GNP_2% and EL_GNP_4%). After 60 d incubation (end time for in vitro degradation test) no further increase in DMEM absorption was observed and its value reached a plateau. Moreover, Figure 5a displays that the fluid uptake pattern was influenced by GNPs content in electrospun matrices, as it was faster in neat mats and for those matrices bearing low GNPs content. This can be explained by the increased hydrophobicity achieved with higher GNPs concentration.

After fluid uptake was determined, the samples were washed three times in order to remove any residues of culture medium inside the matrices. Afterward, the samples were freeze-dried overnight (−48 °C, 0.2 bar) and weighed to determine the mass loss (Equation (3) in Section 2.6.1). The matrices mass essentially remained identical up to 15 d. After 21 d, a mass loss value lower than 4% was found for all the electrospun mats and it reached 15% prolonging the incubation time to 60 d. No significant differences were highlighted depending on GNPs concentration.

Figure 5b shows the SEM images of the matrices after 21 d and 60 d incubation. Morphologic analysis displayed homogeneous fibrous matrices with fibers of about 1 μm diameter and good porosity, for all the samples tested; as previously observed, a variability in fiber diameter distribution was revealed because of different incorporation of the graphene into the polymer matrices. At 60 d incubation, the electrospun matrices showed merged zones with loose texture, whereas GNPs were always visible along polymeric fibers (yellow arrows, Figure 5b).

**Figure 6:**
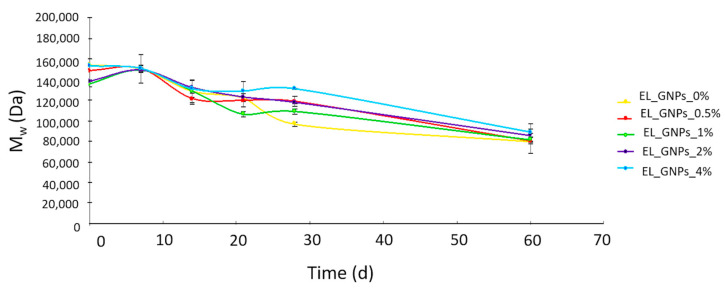
Changes of molecular weight (M_w_, Da) of neat EL_GNP_0% and EL_ GNPs composite matrices incubated with DMEM (10% FBS, 1% antibiotics) at 7, 14, 21, 28 and 60 d.

Figure 6 reports PLA–PCL M_w_ changes along in vitro degradation study. All the tested samples showed a superimposable degradation profile: after 7 d of incubation, the M_w_ remained unchanged with respect to its initial value, between 135–155 kDa; at the 14th d of incubation, M_w_ reduction was between 5% and 18% for all samples. Subsequently, prolonging the incubation up to 21 and 28 d, the average M_w_ reduction was 18% ± 4% and 21% ± 9%, respectively. The slow M_w_ reduction allows us to hypothesize a random hydrolytic cleavage mechanism; a very slow phenomenon that characterizes polymers degrading in bulk such as PLA–PCL.

Finally, at the last timepoint (60 d), there was a significant reduction in polymer M_w_ between the 38% and 48% and M_w_ decreased to 80–88 kDa. The remarkable M_w_ reduction, occurring after 28 d, could be ascribed to backbiting phenomenon that plays an increasingly predominant role as the degradation process proceeds with hydrolytic cleavage [44].

The exponential relationship between M_w_ and degradation time for biodegradable polyesters degrading under *bulk* degradation mechanism was used to compare the degradation rate of the different electrospun mats through Equations (4) and (5) [44,45]:

lnM_w_ = lnM_w0_ − K_Mw_ × t (4)

t_1/2_ = ln2/K_Mw_ (5)

where M_w_ is the weight-averaged molecular weight, M_w0_ is the initial weight-averaged molecular weight, K_Mw_ is the apparent degradation rate and t_1/2_ is the half degradation time. The progress of lnM_w_ was plotted versus the degradation time. The values of K_Mw_ and t_1/2_ were calculated from the slope of the fitting curve during the first 60 d of study (R^2^ > 92%) and reported in Table 3. K_Mw_ values obtained for EL_GNP_0%, EL_GNP_0.5%, EL_GNP_1%, EL_GNP_2% and EL_GNP_4% were 0.0116, 0.0105, 0.0101, 0.0090 and 0.0089, respectively and the corresponding t_1/2_ were 59, 66, 68, 77 and 78 d (Figure 6). Dominating degradation mechanism is random chain scission for high molecular weight polyesters that is a slower mechanism than the end-chain scission one. This evidence is already fully reported in literature however it is difficult to compare different in vitro degradation studies because of the high number of factors affecting the test (e.g., polymer molecular weight, copolymer composition and specimen features) [42]. In this present work, GNPs concentration demonstrated a pivotal role in regulating the samples degradation rate, since t_1/2_ significantly increased by increasing GNPs concentration. GNPs demonstrated to have both roles of enhancer of PLA–PCL mats hydrophobicity and nucleation of polymer crystallization. Polymers chains are more packed in the crystalline domains and water penetration in the mats texture is slowed down. Thus, the crystalline domains with hydrophobic GNPs were more resistant to degradation than amorphous region of copolymer.

Presence of cytotoxic products in the incubation medium of in vitro degradation test was also evaluated through pH monitoring and cell viability determination. Table 4 lists the pH values of sample extracts at 7, 14, 21, 28 and 60 d incubation.

pH determination is a marker measuring polymer degradation since its degradation products are acidic. However, being the culture medium a buffer solution only in case of massive polymer degradation a pH decrease would have been detected. As shown in Table 3, pH value of all extracts was in the range of 8.0–8.5, and no dramatic changes were observed with respect to the initial one, indicating that only a small amount of acid oligomers has been released and the buffering power of the culture medium (with sodium bicarbonate buffer (3.7 g/L)) was able to neutralize. The slight increase of pH was probably caused by the L-Glutamine degradation and the subsequent ammonia accumulation in the degradation media [46,47].

**Figure 7:**
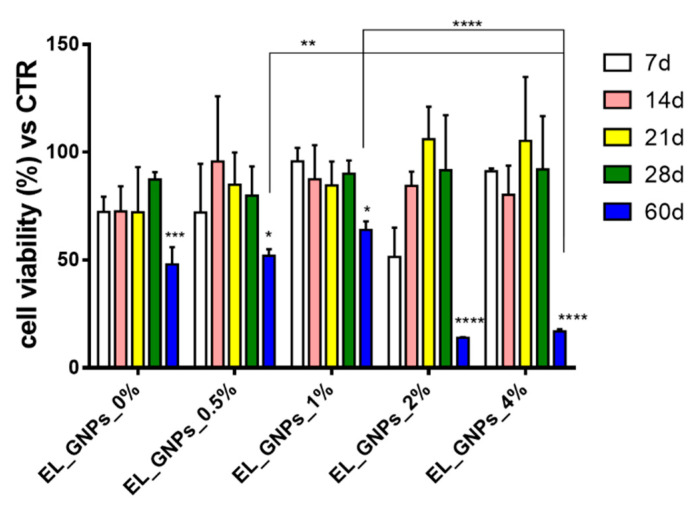
Cytotoxicity evaluation of incubation medium from in vitro degradation test, withdrawn at 7, 14, 21, 28 and 60 d of incubation. Results expressed as cell viability percentage, mean ± SD (*n* = 3). Positive CTR was untreated cells (100% cell viability). Tukey’s multiple comparisons test reveals statistic differences for (*) *p* value < 0.05, (**) < 0.01, (***) < 0.001, (****) < 0.0001.

Figure 7 shows the results in terms of cell viability of NHDFs incubated with the medium withdrawn from in vitro degradation test for 24 h at 37 °C (5% CO_2_).

At the 21st and 28th d of incubation, cell viability was higher than 70% for all samples, thus indicating no toxic degradation products had been released by the electrospun matrices at least till 28 d of incubation. This hypothesis was corroborated by the maintained integrity of the matrices. Cell viability was significantly reduced when cells were in contact with the medium withdrawn after 60 d incubation. Indeed, a significant difference in cell viability results was highlighted depending on sample compositions. The samples coming from incubation medium of neat EL_GNP_0% and samples with the lowest concentrations of GNPs (EL_GNP_0.5%, EL_GNP_1%) revealed a cell viability of 48% ± 8%, 52% ± 3% and 64% ± 4%, respectively, while the EL_GNP_2% and EL_GNP_4% showed the minimum cell viability values for this timing, 14% ± 0.2% and 17% ± 1%, respectively.

### 3.5. Mechanical Properties

**Figure 8:**
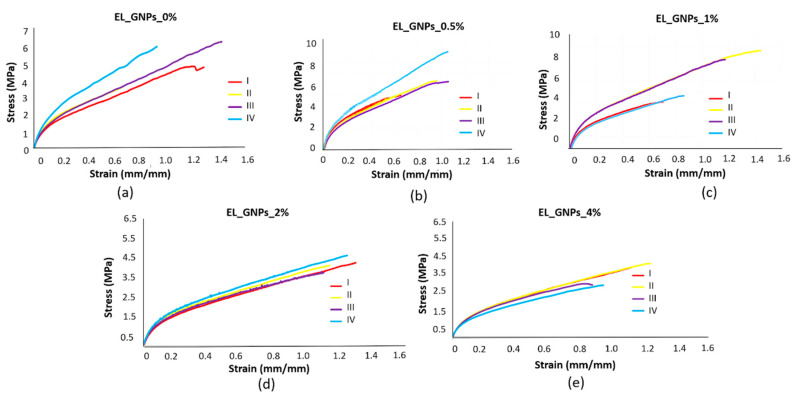
Stress strain curve of: (**a**) EL_GNPs_0%, (**b**) EL_GNPs_0.5%, (**c**) EL_GNPs_1%, (**d**) EL_GNPs_2% and (**e**) EL_GNPs_4%. (I–IV, curves from each dog-bone shaped specimen per mat).

**Figure 9:**
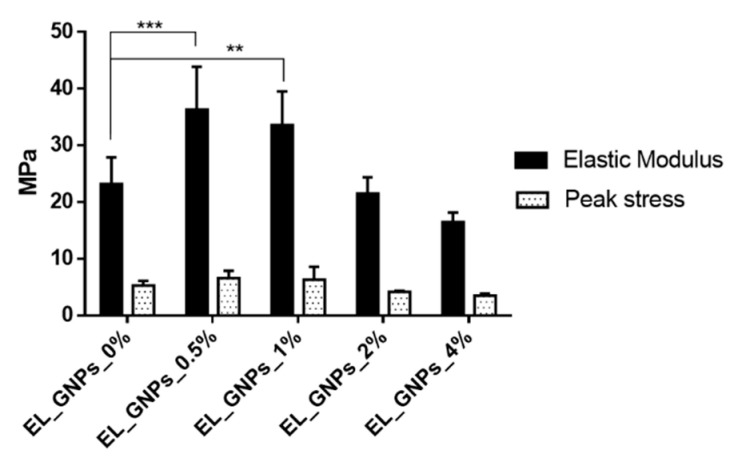
Elastic modulus and peak stress of neat EL_GNPs_0% and composite EL_GNPs matrices. Results are expressed as mean ± SD (*n* = 3). Tukey’s multiple comparisons test reveals statistic differences for (**) *p* value < 0.01, (***) < 0.001.

Neat and composite electrospun samples (suitable thickness about 0.09–0.15 mm) were undergone mechanical tensile tests. The following items were evaluated: (i) stress–strain curves (Figure 8), (ii) elastic modulus and maximum load (Figure 9).

As can be noted for Figure 8, stress–strain curves with a lower dispersion of the data were obtained for matrices containing high GNPs concentrations with respect the copolymer (EL_GNPs_2%–4%). This suggest that the addition of GNPs into the polymeric mats favors reproducible mechanical properties in the whole fibrous mat.

Figure 9 shows the peak stress and elastic modulus values of the electrospun matrices (*n* = 3) expressed as mean ± SD of all the specimens. It is interesting to note how samples containing the lowest GNPs loadings (EL_GNPs_0.5%, EL_GNPs1%) showed the highest peak stress values if compared to the other fibrous matrices examined; in particular, the uppermost peak stress value was found for EL_ GNPs_0.5% (6.6 ± 1.3 MPa). Samples with the highest GNPs concentrations, EL_GNPs_2% and EL_GNPs_4%, presented peak stress values comparable to neat EL_GNPs_0%. Similar behavior was observed for elastic modulus: EL_GNPs_0% and EL_ GNPs_2% samples showed comparable elastic modulus values (23.2 ± 4.7 MPa and 20.6 ± 1.2 MPa, respectively), while EL_ GNPs_4% reached the lowest value of 16.5 ± 1.7 MPa. Conversely, EL_ GNPs_0.5% and EL_GNPs_1% achieved the maximum elastic modulus values of 36.3 ± 7.6 MPa and 33.6 ± 5.9 MPa, respectively.

### 3.6. Thermal Conductivity

GNPs enriched PLA–PCL electrospun mats showed good thermal properties. Excellent results were obtained for the electrospun PLA–PCL-based mats loaded with the highest amount of GNPs (EL_GNP_4%) that exhibited thermal conductivity of 1.27 ± 0.008 W/m K and thermal diffusivity of 1.07 ± 0.068 mm^2^/s. It is worth noting that thermal conductivity value was significantly improving after the GNPs addition compared to neat EL_GNPs_0% matrix (about 0.3 W/m K).

This evidence was also documented in literature for PLA/PCL blend-based composites with carbon nanofillers obtained by compression molding [48].

The data confirmed that electrospinning process led to properly incorporate GNPs along PLA–PCL-based microfibers axes as induced by the strong binding among the two-dimensional (2D) graphene sheets and the thermoplastic polymer matrix. The result was further confirmed by morphologic characterization, see Section 3.2. Moreover, it is also important to highlight that the thermal conductivity of electrospun PCL–PLA based matrices with a GNPs amount of 4 wt % is two-fold higher than that exhibited by PLA-based 3D-printed systems obtained by fusion deposition modeling of PLA-GNPs composite filaments containing a three-fold higher GNPs concentration (12 wt %) [49].

## 4. Conclusions

In this work, PLA–PCL composite mats were successfully fabricated by electrospinning and they were loaded with GNPs ranging from 0.5 wt % to 4 wt %. The addition of GNPs significantly affected physicochemical properties of the electrospun matrices in terms of broader fiber diameter distribution, cold crystallization temperature and polymer crystalline domains. The modulation of electrospun matrices mechanical properties depended on the amount of GNPs loaded. At the same time, the electrospinning process affected GNPs distribution inside the polymer fibers, promoting their base plane orientation to jet flow direction. This last impacted the thermal behavior of the composite electrospun matrices with its significant enhancement.

Eventually, revealed benefits of the reinforced EL_GNPs mats on biologic were pointed out by: (*i*) optimal cells attachment capability and their proliferation on the fibrous matrices and (*ii*) good compatibility of the degradation products released during matrices incubation in physiological environment.

## Figures and Tables

**Table 1:** Samples codes, graphene nanoplatelets (GNPs) content (wt %) and representative images of the electrospun matrices.

Mats Code	GNPs Content (wt %)	Specimens
EL_GNPs_0%	-	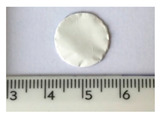
EL_GNPs_0.5%	0.5	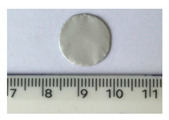
EL_GNPs_1%	1	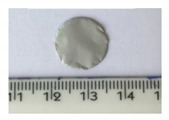
EL_GNPs_2%	2	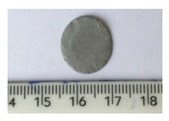
EL_GNPs_4%	4	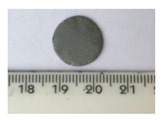

**Table 2:** Thermal data from differential scanning calorimetry (DSC) measurements (second heating) for neat and composite matrices (*n* = 3).

Samples	T_cc_ (°C)	T_m_ (°C)	ΔH_cc_ (J/g)	ΔH_m_ (J/g)
EL_GNP_0%	66.24 ± 0.33	156.20 ± 0.29	9.00 ± 0.47	26.13 ± 1.13
EL_GNP_1%	72.53 ± 0.40	154.27 ± 0.11	11.98 ± 0.56	22.14 ± 1.01
EL_GNP_2%	64.79 ± 0.31	154.16 ± 0.35	9.50 ± 0.51	22.48 ± 0.89
EL_GNP_4%	63.69 ± 0.60	154.33 ± 0.16	6.32 ± 0.44	21.28 ± 0.79

T_cc_ = Cold crystallization temperature, T_m_ = Melting temperature, ΔH_cc_ = Cold crystallization enthalpy, ΔH_m_ = Melting enthalpy.

**Table 3:** Apparent degradation rate (K_Mw_) and half degradation time (t_1/2_) calculated by plotting lnM_w_ vs. degradation time.

Samples	K_Mw_	t_1/2_ (days)	R^2^
EL_GNPs_0%	0.0116	59.75	0.92
EL_GNPs_0.5%	0.0105	66.01	0.95
EL_GNPs_1%	0.0101	68.62	0.94
EL_GNPs_2%	0.0090	77.02	0.94
EL_GNPs_4%	0.0089	77.88	0.95

**Table 4:** pH measurement of incubation medium from in vitro degradation test withdrawn at 7, 14, 21, 28 and 60 d of incubation. Results expressed as mean ± SD (*n* = 3).

Samples	Incubation Time				
	7 d	14 d	21 d	28 d	60 d
EL_GNP_0%	8.15 ± 0.03	8.18 ± 0.02	8.27 ± 0.02	8.21± 0.07	8.29 ± 0.03
EL_GNP_0.5%	8.04 ± 0.05	8.01 ± 0.01	8.30 ± 0.10	8.06 ± 0.06	8.31 ± 0.10
EL_GNP_1%	8.01 ± 0.02	8.04 ± 0.01	8.26 ± 0.04	8.05 ± 0.03	8.20 ± 0.03
EL_GNP_2%	8.09 ± 0.01	8.11 ± 0.07	8.36 ± 0.05	8.12 ± 0.04	8.37 ± 0.02
EL_GNP4%	8.07 ± 0.02	8.07 ± 0.04	8.30 ± 0.03	8.12 ± 0.07	8.41 ± 0.05
